# Fibroblast growth factor 23 inhibition attenuates steroid-induced osteonecrosis of the femoral head through pyroptosis

**DOI:** 10.1038/s41598-024-66799-z

**Published:** 2024-07-15

**Authors:** Lun Fang, Gang Zhang, Yadi Wu, Hao Li, Zhongzhe Li, Beilei Yu, Bin Wang, Lu Zhou

**Affiliations:** 1 Institute of Sports Medicine, College of Sports Medicine and Rehabilitation, Shandong First Medical University & Shandong Academy Medical Sciences, 619 Changcheng Road, Taian, 271016 Shandong People’s Republic of China; 2 Department of Orthopedics, The Second Affiliated Hospital of Shandong First Medical University, Taian, 271000 Shandong People’s Republic of China; 3 School of Laboratory Animal & Shandong Laboratory Animal Center, Shandong First Medical University & Shandong Academy of Medical Sciences, Jinan, 250117 Shandong People’s Republic of China; 4 Medical School of Nanjing University, Nanjing University, Nanjing, 210093 Jiangsu People’s Republic of China

**Keywords:** Osteonecrosis of the femoral head, Steroid, FGF23, Pyroptosis, Cell biology, Molecular biology

## Abstract

Steroid-induced osteonecrosis of the femoral head (SONFH) is the predominant cause of non-traumatic osteonecrosis of the femoral head (ONFH). Impaired blood supply and reduced osteogenic activity of the femoral head are the key pathogenic mechanisms of SONFH. Fibroblast growth factor 23 (FGF23) levels are not only a biomarker for early vascular lesions caused by abnormal mineral metabolism, but can also act directly on the peripheral vascular system, leading to vascular pathology. The aim of this study was to observe the role of FGF23 on bone microarchitecture and vascular endothelium, and to investigate activation of pyroptosis in SONFH. Lipopolysaccharide (LPS) combined with methylprednisolone (MPS) was applied for SONFH mouse models, and adenovirus was used to increase or decrease the level of FGF23. Micro-CT and histopathological staining were used to observe the structure of the femoral head, and immunohistochemical staining was used to observe the vascular density. The cells were further cultured in vitro and placed in a hypoxic environment for 12 h to simulate the microenvironment of vascular injury during SONFH. The effect of FGF23 on osteogenic differentiation was evaluated using alkaline phosphatase staining, alizarin red S staining and expression of bone formation-related proteins. Matrigel tube formation assay in vitro and immunofluorescence were used to detect the ability of FGF23 to affect endothelial cell angiogenesis. Steroids activated the pyroptosis signaling pathway, promoted the secretion of inflammatory factors in SONFH models, led to vascular endothelial dysfunction and damaged the femoral head structure. In addition, FGF23 inhibited the HUVECs angiogenesis and BMSCs osteogenic differentiation. FGF23 silencing attenuated steroid-induced osteonecrosis of the femoral head by inhibiting the pyroptosis signaling pathway, and promoting osteogenic differentiation of BMSCs and angiogenesis of HUVECs in vitro.

## Introduction

Osteonecrosis of the femoral head (ONFH) is one of the common clinical diseases in orthopedics. The main clinical features are limited flexion and extension, claudication, severe pain in the hip and so on. It has a high prevalence in young and middle-aged people aged 20–50 years old, with a trend towards younger age^1^. There is a high disability rate in ONFH, which seriously affects patients' physical health and quality of life, and brings great psychological pressure to patients. ONFH is divided into non-traumatic and traumatic, and steroid-induced osteonecrosis of the femoral head (SONFH) is the main cause of non-traumatic osteonecrosis of the femoral head. The "vascular theory" plays a leading role in the pathophysiological mechanism of SONFH and is gradually being recognized^2^. The imbalance in the vascular homeostasis of the femoral head causes an impaired blood supply to the femoral head, resulting in hypoxia of the corresponding tissues, which can lead to programmed death and damage of osteoblasts and consequently to the collapse of the femoral head structure^3^. New research has shown that the treatment of ONFH by inhibiting inflammatory factors and protecting vascular endothelial cells from damage has been effective in animal models and in clinical practice^4,5^.

Fibroblast growth factor 23 (FGF23) is a phosphophilic hormone produced by bone. Studies have shown that FGF23 is a physiological regulator of phosphate and vitamin D metabolism and is indispensable for maintaining serum phosphate levels^6^. FGF23 has been identified as a gene associated with autosomal dominant hypophosphatemic rickets (ADHR), which plays an important role in the development of bone diseases^7,8^. As research progressed, several reports identified different roles for FGF23 in metabolic regulation, rheumatism, and cardiac hypertrophy. In particular, FGF23 promotes reactive oxygen species (ROS) production by upregulating the expression of reductive coenzyme II oxidase 2 in coronary artery endothelial cells, and influences cell apoptosis and vascular injury by stimulating endothelial cell migration and proliferation^9,10^.

In recent years, the study of SONFH has penetrated into the cellular biochemical level, which mainly includes apoptosis, autophagy^11^, pyroptosis. Among them, pyroptosis is a new mode of cellular inflammatory and programmed death between apoptosis and necrosis, which is a programmed cellular death mediated by gasdermin (GSDMs) with the involvement of NOD-like receptor protein 3 (NLRP3) inflammatory vesicles and dependence on cysteinyl aspartate specific proteinase (caspase). Cells suffer from external stimuli through a series of immune responses to initiate pyroptosis, followed by swelling, rupture of the cell membrane and death, and at the same time release a large amount of inflammatory substances accumulated in the damaged area and thus inflammatory response occurs^12^. Cellular pyroptosis is widespread in eukaryotic organisms and is a form of cellular self-protection against external damage, but over-activation can lead to organismal damage. Steroid use can activate bone marrow mesenchymal stem cells (BMSCs), which is closely related to the development of SONFH.

Therefore, this study was conducted to investigate the progression of ONFH in the case of FGF23 interference with avascular necrosis, with the intention of exploring the role of FGF23 in ONFH disease and related mechanisms, and providing a viable clinical treatment basis for the early prevention and treatment of SONFH.

## Materials and methods

### Adenovirus production

FGF23-overexpressing (FGF23-overexpressing) recombinant adenovirus (ad-FGF23), blank control recombinant adenovirus (LacZ) and FGF23-silenced recombinant adenovirus were purchased from Hanheng Biotechnology Co., Ltd. Human FGF23 cDNA was packaged into adenoviral vectors. The virus was amplified in human embryonic kidney 293 (HEK293) cells (ATCC, CRL 1573) and purified by Vivapure Adeno PACK 20 (Sartorius). Steroid-induced osteonecrosis of the femoral head was performed in SD rats 24 h after transfection with ad-FGF23, LacZ, and si-FGF23 according to the manufacturer’s instructions.

### Animals and SONFH models

A total of 75 SPF-grade SD rats (7–8 weeks old, weight 250 g ± 20 g) were provided by the Experimental Animal Centre of Shandong First Medical University. The rats were randomly divided into the following five groups: blank control group (NC), model control group (con), LacZ group (LacZ), FGF23 overexpression group (ad-FGF23) and FGF23 silencing group (si-FGF23). Firstly, lipopolysaccharide (LPS, 10 μg/kg) was injected through the tail vein. 24 h later, methylprednisolone (MPS, 20 mg/kg) was injected intramuscularly in three times with a 24 h interval^13^. While the NC group was injected with the same amount of saline. On the day before modeling, 0.1 mL/10 g adenovirus (1 × 108 IU or 2 × 108 IU) was injected into the tail vein of rats in the LacZ, ad-FGF23, and si-FGF23 groups, respectively, to increase or decrease the corresponding protein levels. The LacZ virus served as a functional control for adenovirus. The rats were sacrificed for histological and biochemical analysis after 8 weeks of adenovirus. This study has been approved by the Animal Ethics Committee of Shandong First Medical University (Shandong Academy of Medical Sciences), approval number: No.W202210070244. All experiments were conducted in accordance with the guidelines outlined in the Association for Research in Vision and Ophthalmology (ARVO) Statement. The study was carried out in compliance with the ARRIVE guidelines.Micro-computed tomography (micro-CT).

After completion of the SONFH model, the femur was collected, the surrounding muscles and other soft tissues were removed, and the femoral specimen was fixed in 4% paraformaldehyde and scanned by micro-CT. Key trabeculae parameters were identified, including bone volume (BV, mm^3^), bone volume fraction (BV/TV), number of trabeculae (Tb.N, 1/mm) and trabecular separation (Tb.Sp, mm).

### Histological staining

Femurs were fixed in 4% paraformaldehyde (cat.no.G1101; Servicebio), decalcified in 10% ethylenediaminetetraacetic acid (EDTA; cat.no.G1105; Servicebio) decalcifying solution for 4 weeks, and then embedded in paraffin. The tissue was sectioned longitudinally, cut into 5-μm-thick sections and stored at room temperature for use. Hematoxylin–Eosin (HE; cat.no.G1005; Servicebio) and Safranin O-Fast Green staining (cat.no.G1053; Servicebio) was performed. Finally, the sections were sealed with central gum, placed under a light microscope and photographed for analysis.

### Enzyme linked immunosorbent assay (ELISA)

Serum of mice and cell culture medium from each group were collected. And ELISA kits were used to measure IL-1β (cat.no.ml003057; mlbio), IL-6 (cat.no.ml064292; mlbio), TNF-α levels (cat.no.ml002859; mlbio), FGF23 (CSB-E12170r, CUSABIO), VEGF-A (RK00066, Abclonal).

### Immunohistochemical staining

Sections were dewaxed in xylene and hydrated in gradient ethanol for 5 min each, followed by antigen recovery. Sections were performed in EDTA antigen retrieval solution under microwave high fire, incubated in 3% H_2_O_2_ at 37 °C for 15 min, washed with phosphate buffer saline (PBS; cat.no.G4202-500 ML; Servicebio) and blocked with 3% bovine serum albumin (BSA; cat.no.A8020; Solarbio) at room temperature for 30 min, incubated with anti-CD31 (1:1000; cat.no.3528; Cell Signaling Technology, Inc.), anti-VEGF (1:1000; cat.no.9698; Cell Signaling Technology, Inc.) antibodies overnight at 4 °C. The sections were then incubated with secondary antibodies at room temperature for 1 h. They were developed with 3,3-diaminobenzidine tetrahydrochloride, then re-stained with hematoxylin for 3 min, dehydrated and transparent, and sealed. Observation under the microscope.

### Isolation and culture of mouse bone marrow stromal cells (BMSCs)

Male C57BL/6 J mice of 3–4 weeks of age during the growth period were selected. The cervical vertebrae were dislocated and sterilized by immersion in 75% ethanol for about 5 min. The femur and tibia were separated and washed several times in sterilized 1 × PBS containing 100 μg/ml streptomycin and 100 U/ml penicillin (Sigma-Aldrich, St Louis. MO, USA). The epiphyses of the two ends were cut to make the bone marrow cavity in an open state, The bone marrow cavity was rinsed with dulbecco's modified eagle medium (DMEM; Lot.8121218; Gibco) and the rinsate was collected, centrifuged at 1000 rpm/min for 5 min, the supernatant was discarded, and resuspended by adding DMEM complete culture medium, the cell suspension was inoculated in a constant temperature incubator at 37 ℃, with a volume fraction of 5% CO_2_, and the growth of the cells was observed under the microscope. Replace the complete culture medium after 2 days, wait until the cells are confluent to 80–90% for passaging, and then keep changing the medium 2–3 days and passaging culture in time. The third generation of cells was used for all subsequent experiments.

### Cell model

Human umbilical vein endothelial cells (HUVECs) were from American Type Culture Collection (ATCC, PCS-100-013). BMSCs and HUVECs were cultured in Oxoid AnaeroGen anaerobic tanks (cat. no. HBYY001; hopebio) with the aim of simulating the ischaemic-hypoxic microenvironment of vascular injury during SONFH in vitro. The anaerobic capsules in the sealed jars will rapidly absorb atmospheric oxygen and produce CO_2_, eventually bringing the oxygen concentration to less than 1% within 30 min^14^.

### Cell transfection and grouping

Logarithmically grown BMSCs and HUVECs were inoculated into 6-well plates at a cell density of 2 × 10^5^ per well and transfected when cells were fused to 30–50%. The adenovirus were synthesized by HANBIO (Hanheng Biological, Lot.xbd-001). LacZ adenovirus, ad-FGF23 adenovirus and si-FGF23 adenovirus (MOI 50) were added dropwise to serum-free, antibiotic-free medium. Cells without treatment were also used as a control group. The medium was replaced with fresh complete medium after 6 h of transfection. Cells were divided into the following five groups: normal control group (NC), hypoxia model control group (con), adenovirus functional control group (LacZ), FGF23 overexpression group (ad-FGF23) and FGF3 silencing group (si-FGF23).

### Alkaline phosphatase (ALP) staining and Alizarin red staining (ARS)

BMSCs were seeded in 6-well plates at a cell density of 5 × 10^4^. When the cells were grown to 80%, the culture medium was replaced with osteogenic induction medium (complete medium containing 10 mM sodium β-glycerophosphate (cat.no. A56289; OKA) and 50 μg/ml ascorbic acid) to induce differentiation into mature osteoblasts^15^. After 7 days of induction, alkaline phosphatase activity was measured by ALP staining kit (cat.no.C3206; Beyotime Biotech Inc). After 21 days of induction, the mineralized nodule formation characteristics of BMSCs were measured by ARS (cat.no. G1452; Solarbio).

### Tube formation assay in vitro

Matrigel (cat.no.0827045; ABW) was diluted with DMEM at a ratio of 1:3 and seeded to a 96-well plate. 50μL matrigel was added to each well. HUVECs were inoculated at a density of 3 × 10^4^ in the 96-well plate and continued to be cultured for 8 h. HUVECs were observed under a light microscope to see if they formed a tubular lumen-like structure^16^.

### Immunofluorescence

HUVECs were fixed in 4% paraformaldehyde solution (cat.no.P1110; Solarbio) for 30 min, permeabilised with 0.5% Triton X-100 (cat.no.T8200; Solarbio) for 10 min, and then blocked with 3% BSA (cat.no.A8020; Solarbio) at room temperature for 30 min. Subsequently, HUVECs were incubated with anti-VEGF (1:1000; cat.no.9698; Cell Signaling Technology, Inc.), γ-H2aX (cat.no.2035S; Beyotime) antibody and BMSCs were incubated with anti-Runx2 (1:500; cat.no.GB115631; Servisebio) and anti-α-Tubulin (1:500; cat.no.ab179484; Abcam) antibody overnight at 4 °C and then incubated with secondary antibody at room temperature for 1 h. Cell nucleus were stained with DAPI (0.5 μg/mL; cat.no.C0060; Solarbio). Samples were observed under a fluorescent microscope.

### Hoechst 33342/PI fluorescent staining

Pyroptosis was assessed by double staining of cells with Hoechst 33342 and PI^17^. HUVECs were inoculated in 6-well plates in complete medium. After the indicated treatments, cells in each group were stained with staining solution Hoechst 33342 (cat.no.C1027; Beyotime) and 2 µg/mL PI (cat.no.C0080; Solarbio) for 20 min. The cells were then washed three times with PBS. Samples were photographed under a fluorescent microscope.

### Western blot

RIPA lysate (cat.no.R0010; Solarbio) was added to extract the total protein of femoral head and cells. BCA protein assay kit (cat.no.PC0020; Solarbio) was used to measure the protein concentration. 10% separation gel and 5% concentration gel were prepared. The samples were loaded sequentially at 30 μg protein per well. 5% concentrated gel was electrophoresed at 80 V for 30 min, then switched to 120 V for 10% separation gel for 1 h. The membranes were transferred at 100 V for 1 h in an ice bath. Block with 5% skimmed milk (cat.no.D8340; Solarbio) for 1 h. The primary antibodies were diluted with 5% BSA solution at a ratio of 1:1000. Remove the blocking solution and add the corresponding FGF23 (1: 500; cat.no.A6124; Abclonal), Runt-related transcription factor 2 (Runx2; 1:1000; cat. no. 12556; Cell Signaling Technology, Inc.), Osteocalcin (OCN; 1:500; cat.no.A20800; Abclonal), ALP (1: 1000; cat. no. ab229126; Abcam), VEGF (1:1000; cat.no.9698; Cell Signaling Technology, Inc.), NOD-like receptor thermal protein domain associated protein 3 (NLRP3; 1:1000; cat.no.A5652; Abclonal), caspase-1(1:1000; cat.no.A0964; Abclonal), Gasdermin D (GSDMD; 1:500; cat.no.A17308; Abclonal), β-actin (1:5000; cat.no.AB0035; Abways Technology) primary antibody and incubated overnight at 4 °C. The secondary antibody was incubated at room temperature for 1 h. The protein bands were observed using the ECL chemiluminescence kit (cat.no.P10200; New Cell & Molecular Biotech Co., Ltd). β-actin was used as a reference. The original protein images were provided as supplementary figures (Supplementary Information).

### Statistical analysis

The experiments were repeated three times and the data were statistically processed using GraphPad Prism 6.02 software. *t*-test was used to compare only two groups. One-way ANOVA was used to compare differences across several groups. Differences were considered statistically significant at *P* < 0.05.

### Ethical approval

This study was approved by the Research Ethics Committee of Shandong First Medical University (Shandong Academy of Medical Sciences) (grant no. W202210070244).

## Results

### FGF23 exacerbates the destruction of the femoral head by steroids

**Figure 1:**
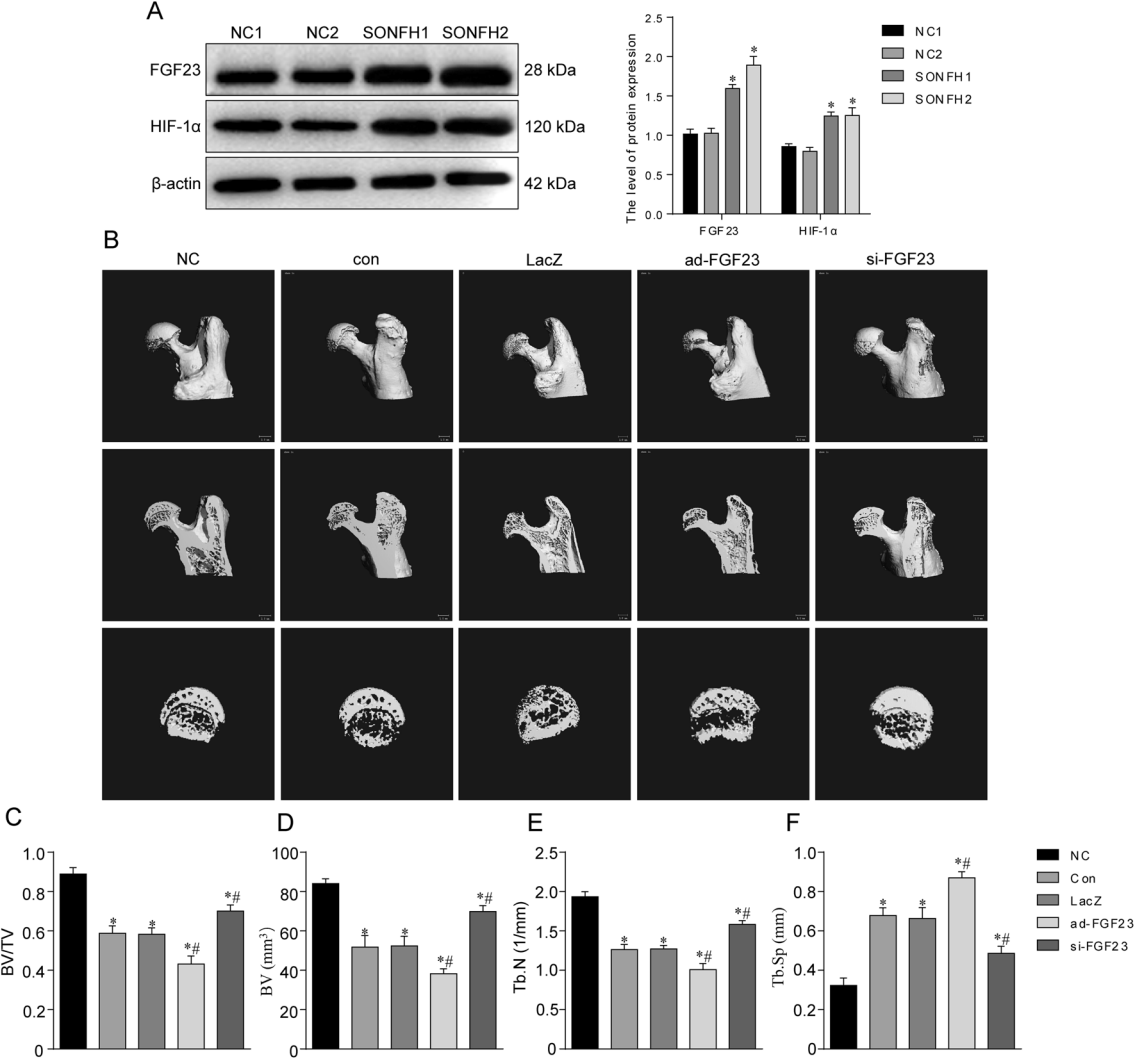
FGF23 exacerbates the damage of steroids to the bone microstructure of the femoral head. (**A**) Western blot detection of FGF23 and HIF-1α expression levels in the femoral head region of the SONFH model. (**B)** Micro-CT scan of the femoral head. (**C**–**F**) Bone trabecular parameters, including BV, BV/TV, Tb.N and Tb.Sp. *P < 0.05 versus NC group. ^#^*P* < 0.05 versus LacZ group.

**Figure 2:**
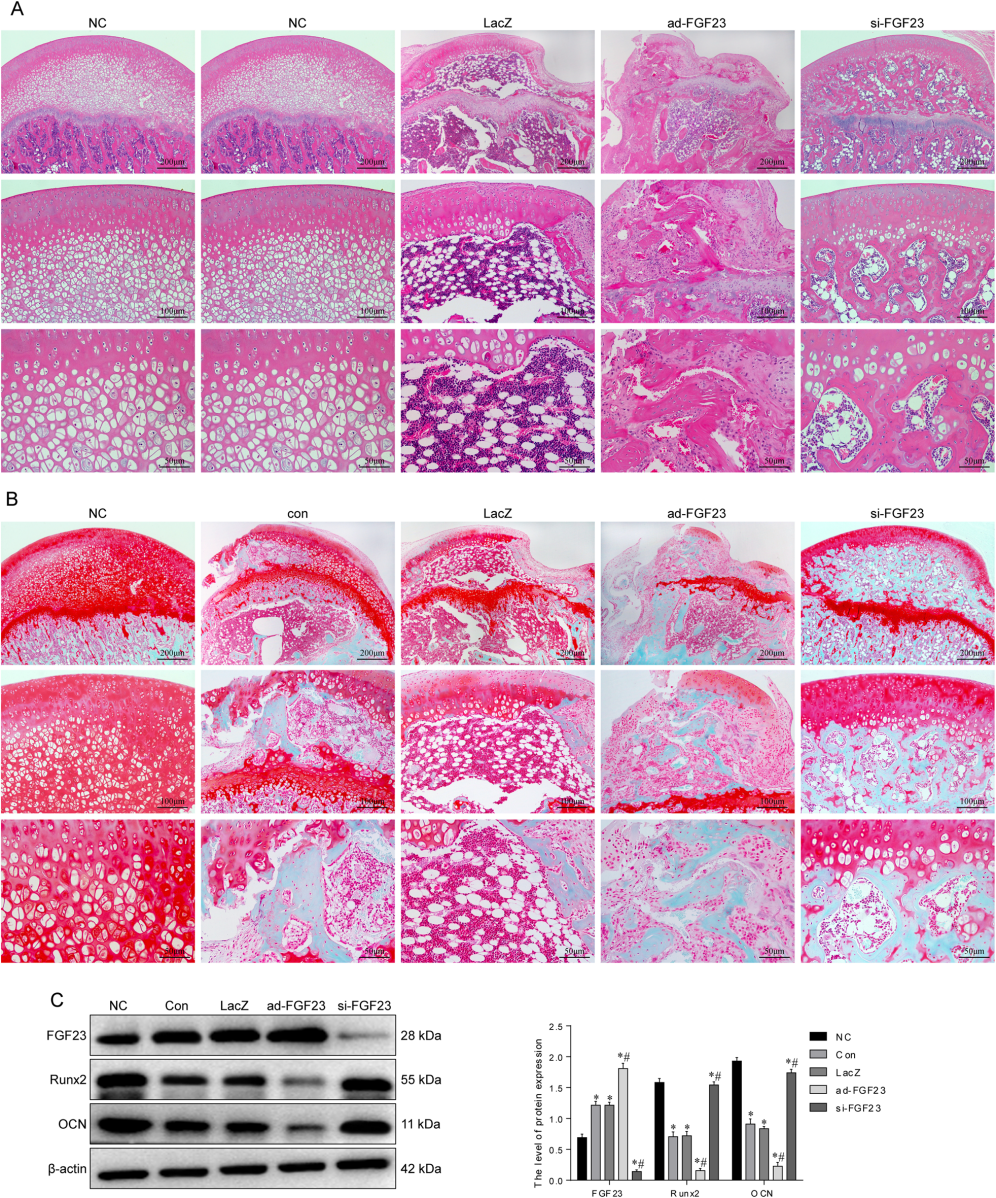
Inhibition of FGF23 reduces the damage of steroids to the structure of the femoral head. (**A**) Representative image of HE staining of the femoral head. (**B**) Representative images of the femoral head stained with Saffran O-Fast Green. (**C**) Western blot to detect the expression levels of bone formation-related proteins in the femoral head. *P < 0.05 versus NC group. ^#^*P* < 0.05 versus LacZ group.

Steroid overuse can damage the blood supply of the femoral head, resulting in tissue hypoxia at the corresponding site. We examined the expression of hypoxia inducible factor-1 alpha (HIF-1α) at the femoral head site, which was upregulated in the model group (Fig. 1A). Our previous study demonstrated that hypoxia led to upregulation of FGF23 expression in osteoblasts. To further verify the changes of FGF23 during the development of SONFH, FGF23 expression levels were found to be elevated in the femoral head necrosis region in SONFH modes by western blot (Fig. 1A). To further investigate the effect of FGF23 on the bone microstructure of the femoral head in steroid-treated mice in vivo, we first overexpressed or silenced FGF23 by tail vein injection of LacZ, ad-FGF23 and si-FGF23 adenovirus. Analysis of the micro-CT results showed that the femoral head structure of the model control mice was distroyed (Fig. 1B), the trabecular gap was enlarged, the bone density, BV, BV/TV, and Tb.N decreased (Fig. 1C–F). This disruption was exacerbated by the overexpression of FGF23. FGF23 silencing resulted in an intact femoral head structure and improved BV, BV/TV, Tb.N and Tb.Sp. Histological staining showed the cartilage layer of the femoral head became thinner and bone trabeculae arranged disorderly after steroids treatment (Fig. 2A,B). Further aggravation of femoral head destruction was observed in the ad-FGF23 group, which was partially reversed by FGF23 silencing. Furthermore, we extracted mice femoral head proteins and the results showed that FGF23 inhibited the expression levels of the bone formation marker genes ALP, Runx2 and OCN (Fig. 2C).

### FGF23 promotes the secretion of inflammatory factors to damage blood vessels

**Figure 3:**
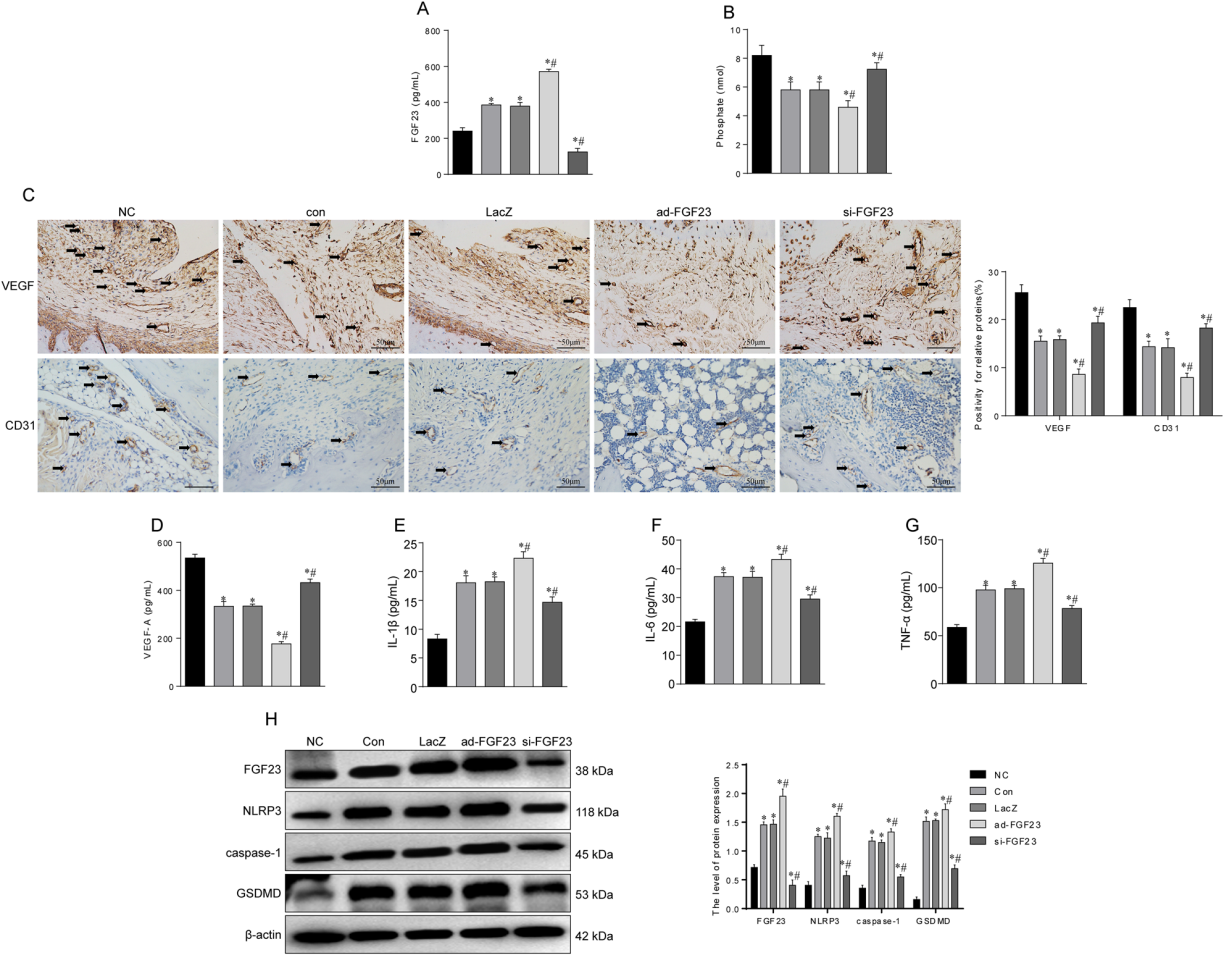
FGF23 promotes the secretion of inflammatory factors that damage blood vessels. (**A**) Circulating FGF23 expression. (**B**) Serum phosphate level. (**C**) Immunohistochemical staining of VEGF and CD31-related antigens and vascular density in the femoral head. (**D**) VEGF-A isoform expression in serum. (**E**–**G**) Levels of inflammatory factors including IL-1β, IL-6, TNF-α in serum of SONFH. (**H**) Western blot to detect the expression levels of proteins related to pyroptosis signaling pathway. *P < 0.05 versus NC group. ^#^*P* < 0.05 versus LacZ group.

In the early stage of SONFH, due to the disturbance of lipid metabolism, a large number of inflammatory factors are secreted, leading to vascular endothelial damage. Moreover, related studies have shown that FGF23 can act directly on the peripheral vascular system, leading to vascular lesions. We examined FGF23 expression (Fig. 3A) and phosphate levels (Fig. 3B) in serum, and increased circulating FGF23 expression was observed in the model group, while phosphate levels in serum decreased at the same time, and phosphate levels decreased as FGF23 expression was upregulated. In the present study, to verify the effect of FGF23 on angiogenesis in the femoral head, VEGF and CD31 were chosen for immunohistochemical staining. The results showed that VEGF and CD31 expression was reduced in the model group and the number of intact microvessels was less (Fig. 3C). While VEGF and CD31-positive cells were increased, the microvascular structure was largely intact and the blood vessel density was increased in the si-FGF23 group. And VEGF-A isoform expression is elevated in serum and promotes angiogenesis (Fig. 3D). In addition, we found that the expression levels of inflammatory factors (IL-1β, IL-6, TNF-α) in the serum of the model group were significantly higher than those in the control group (Fig. 3E–G). Moreover, we extracted femoral proteins and western blot results showed that the expression levels of NLRP3, caspase-1, and GSDMD were upregulated in the model group (Fig. 3H). These results suggest that steroids increase the secretion of inflammatory factors in mice, which may activate the pyroptosis signaling pathway.

### FGF23 overexpression inhibited osteogenic differentiation in vitro

**Figure 4:**
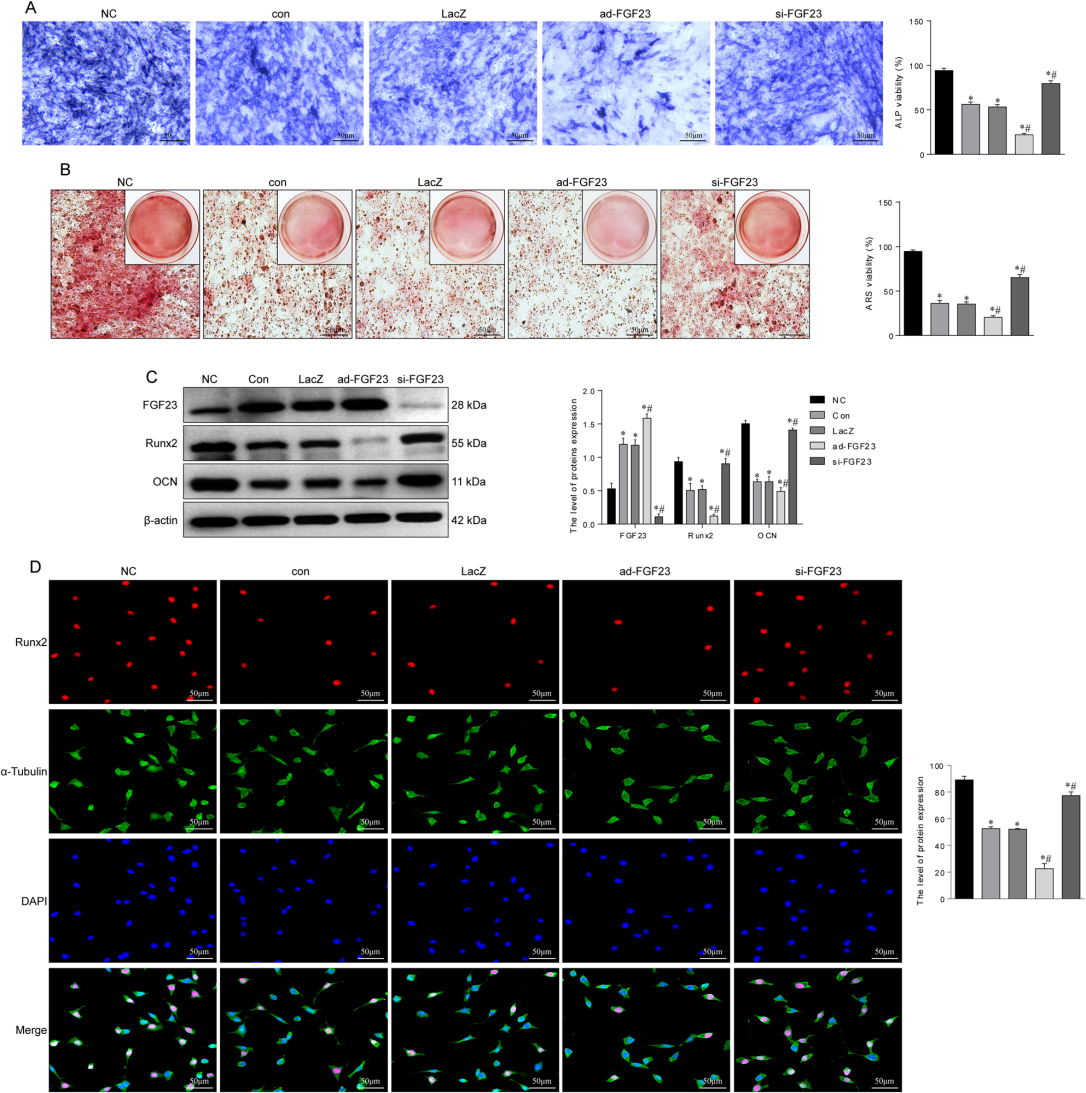
Interference with FGF23 expression affects osteogenic differentiation in vitro. (**A**) ALP staining to detect the activity of ALP in BMSCs 7 days after induction of osteogenic differentiation (**B**) ARS staining to detect the number of mineralized nodules in BMSCs 21 days after induction of osteogenic differentiation. (**C**) Effect of FGF23 on the expression levels of bone formation-related proteins in BMSCs. (**D**) Immunofluorescence staining to detect the expression level of Runx2. *P < 0.05 versus NC group. ^#^*P* < 0.05 versus LacZ group.

Since FGF23 overexpression was found to exacerbate steroids damage to the femoral head structure and to inhibit the expression of bone formation-related proteins, we further assessed whether FGF23 was associated with osteoblast differentiation in vitro. After induction of differentiation, ALP and ARS staining revealed reduced ALP activity and reduced mineralized nodule formation in hypoxia-treated BMSCs compared to the normal group (Fig. 4A,B). ALP is a marker of early osteogenic differentiation and mineralized nodules are a marker of late osteogenic differentiation^18^. In addition, the protein expression levels of the typical osteogenic markers Runx2, ALP and OCN were reduced in the hypoxic group (Fig. 4C). Overexpression of FGF23 further inhibited the osteogenic function of BMSCs. Silencing of FGF23 protected the ALP activity and mineralization properties of BMSCs and increased the expression of osteogenic-related genes. Immunofluorescence results also showed that Runx2 expression was up-regulated in the si-FGF23group (Fig. 4D). These results suggest that FGF23 is essential for osteogenic differentiation.

### FGF23 silencing promotes angiogenesis in vitro

**Figure 5:**
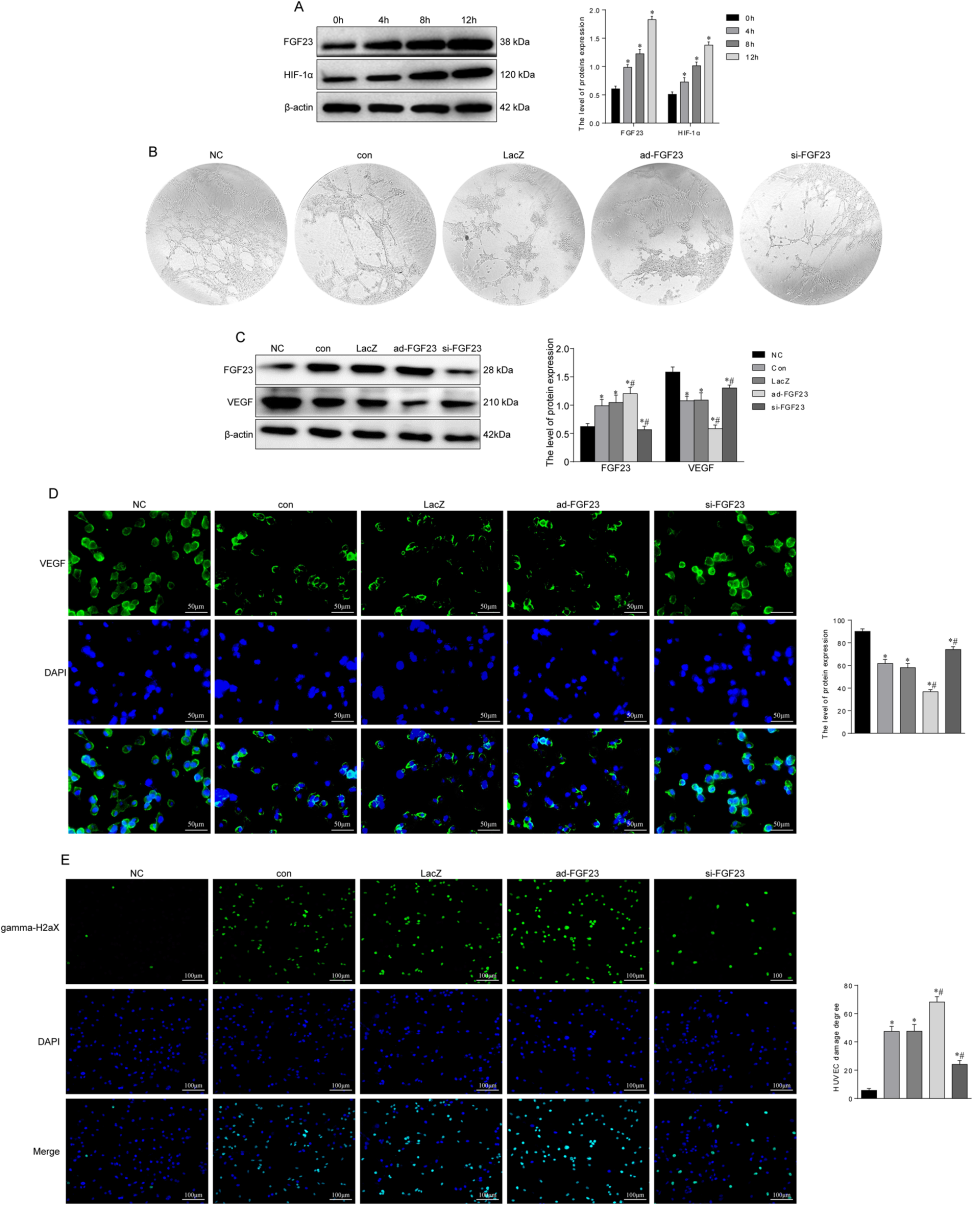
FGF23 silencing promotes angiogenesis in vitro. (**A**) The expression level of FGF23 and HIF-1α in HUVECs after hypoxia was detected by western blot. (**B**)Tube-forming assay in vitro to detect the effect of FGF23 on the formation of official lumen-like structures in HUVECs. (**C**) Effect of FGF23 on VEGF, an angiogenesis-related protein in HUVECs. (**D**) Immunofluorescence staining images of HUVECs after interference with FGF23. (**E**) Gamma-H2aX staining was used to detect DNA damage induced by FGF23 in HUVEC. *P < 0.05 versus NC group. ^#^*P* < 0.05 versus LacZ group.

Experiments in vivo revealed that vascular injury was associated with FGF23 expression. Firstly, HUVECs were placed in a hypoxic environment to mimic the vascular injury microenvironment during SONFH. Western blot results showed that FGF23 expression in HUVECs was upregulated by hypoxia in a time-dependent manner, and the expression reached the highest level at 12 h of hypoxia, so 12 h of hypoxia was selected for subsequent experiments (Fig. 5A). We used tube formation assay to further verify the effect of FGF23 on endothelial cell angiogenesis in vitro (Fig. 5B). The results showed that vascular-like structures were incomplete or sparse in the ad-FGF23 group, whereas endothelial cells in the si-FGF23 group differentiated to form complete circular vessel-like structures. In addition, western blot results showed that FGF23 overexpression inhibited the expression level of VEGF in HUVECs, and FGF23 silencing reversed this result (Fig. 5C). The results of immunofluorescence were consistent with those of western blot (Fig. 5D). Vascular DNA damage was reduced following FGF23 silencing as revealed by gamma-H2aX staining (Fig. 5E).

### FGF23 silencing inhibits pyroptosis signaling pathway

**Figure 6:**
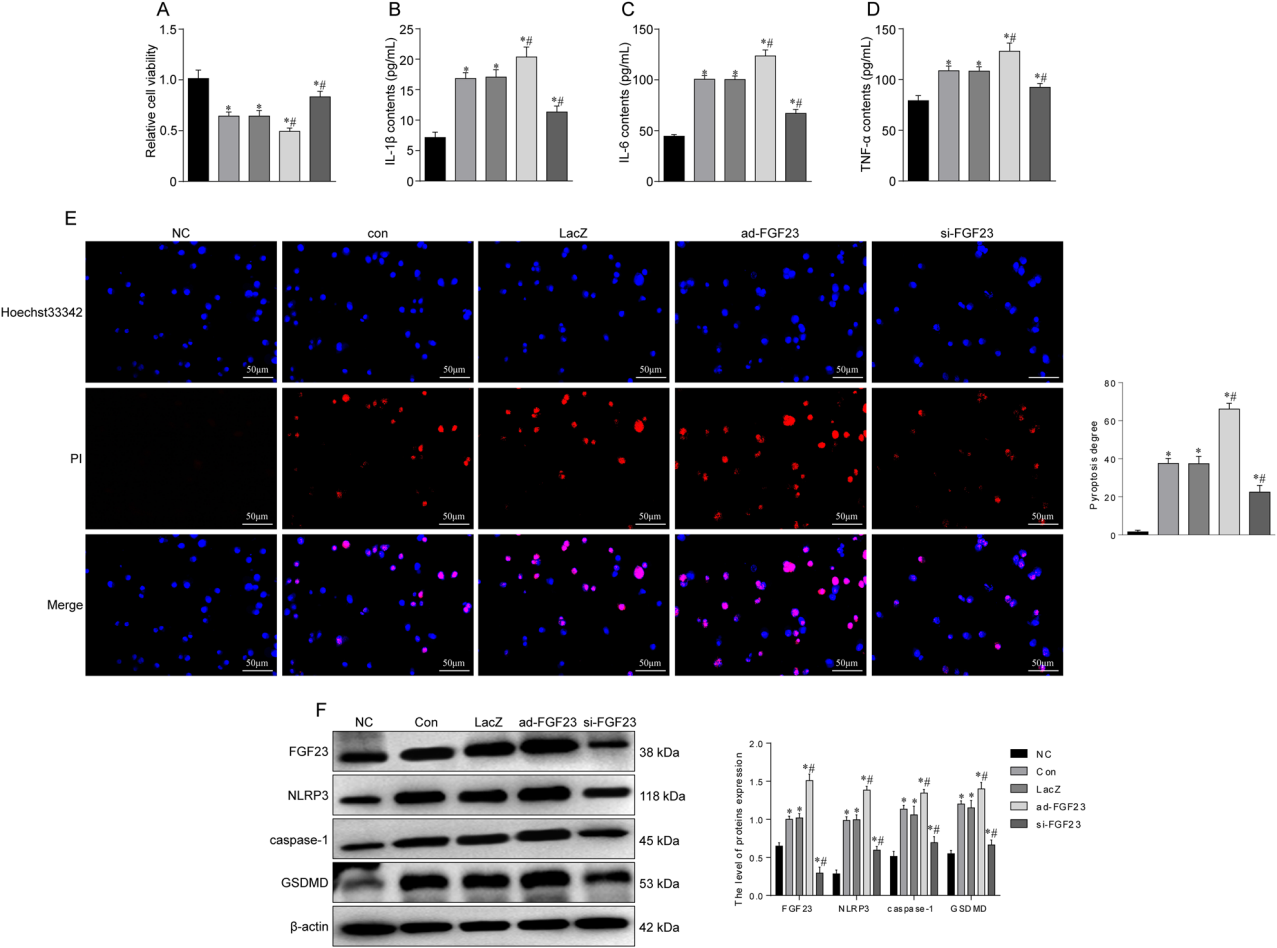
FGF23 regulates the pyroptosis signaling pathway to influence the progression of SONFH. (**A**) MTT assay was used to examine the viability of HUVECs after hypoxia. (**B**–**D**) Levels of inflammatory factors including IL-1β, IL-6, TNF-α in HUVECs after interference with FGF23. (**E**) Hoechst 33342/PI fluorescence staining to detect activation of pyroptosis signaling pathway. (**F**) Western blot to detect expression levels of proteins related to pyroptosis signaling pathway. *P < 0.05 versus NC group. ^#^*P* < 0.05 versus LacZ group.

FGF23 silencing protected the overall activity of HUVECs as revealed by MTT analysis. We then further evaluated the effect of FGF23 on pyroptosis in hypoxia-treated HUVECs after 12 h (Fig. 6A). In addition, FGF23 silencing significantly inhibited the release of IL-1β, IL-6, and TNF-α induced by hypoxia in HUVECs (Fig. 6B–D). Hoechst 33342/PI fluorescence staining showed that FGF23 silencing significantly reduced the number of PI-positive cells in hypoxia-induced HUVECs (Fig. 6E). Furthermore, FGF23 silencing reduced the protein expression of NLRP3, caspase-1 and GSDMD in hypoxia-induced HUVECs (Fig. 6F). These results suggest that FGF23 silencing may partially protect HUVECs from hypoxia-induced endothelial pyroptosis.

## Discussion

In the present study, we validated the role of FGF23 in SONFH. We found that steroids can upregulate FGF23 expression, increase the secretion of inflammatory factors and impair bone microarchitecture and angiogenesis. Our data also suggest that FGF23 silencing can promote osteogenic differentiation and reduce vascular endothelial damage in vitro, thereby preventing the development of ONFH.

In this study, LPS combined with MPS was used to establish animal models of SONFH in mice. We found that the femoral head surface structure was incomplete and the trabecular structure of the subchondral bone area was severely damaged in the model group. The expression of FGF23 was associated with bone formation and bone resorption. In our study, the effect of FGF23 on the bone microarchitecture of SONFH in mice was first assessed by micro-CT and histopathological assays. It was found that FGF23 overexpression significantly reduced the estimation of the femoral head and disrupted the trabecular parameters, while FGF23 silencing improved this outcome. It was found that chronic steroids exposure, which resulted in upregulation of serum FGF23 and bone FGF23 expression in mice, partially reduced longitudinal bone growth, decreased mineral density and led to impaired bone growth^19^. Moreover, studies in vitro have shown that hypoxia inhibits osteoblast differentiation potential, while FGF23 silencing improved ALP activity, increases the number of calcified nodules and improved osteoblast differentiation. In addition, western blot results also showed that the expression of osteogenic markers Runx2, ALP and OCN was upregulated after FGF23 silencing. This finding is consistent with previous studies showing that interference with FGF23 expression can affect osteogenic differentiation, which in turn affects the generation of osteoblasts^20,21^. Therefore, these results suggest that interference with FGF23 expression can regulate osteoblast differentiation to influence bone formation and thus the development of ONFH.

In the early stages of SONFH, much vascular damage occurs due to disruption of lipid metabolism, resulting in increased intravascular pressure and massive secretion of inflammatory factors. The results of the present study also revealed that reduced vascular density was found in the SONFH models, and immunohistochemical results showed the expression of key angiogenic proteins CD31 and VEGF decreased. Related studies have found that FGF23 plays an important role in regulating the secretion of inflammatory factors and damage to the vascular endothelium^22^. In the present study, we also confirmed that FGF23 overexpression inhibited the local angiogenesis of the femoral head in SONFH models, and the protein levels of CD31 and VEGF in bone tissue were significantly downregulated. In addition, we confirmed that FGF23 impairs the function of the vascular endothelium by further studies in vitro. Firstly, FGF23 reduced the overall activity of HUVECs as revealed by MTT analysis. Another report directly demonstrates that FGF23 causes vascular endothelial dysfunction. Tube-forming assays in vitro showed that FGF23 inhibited the differentiation of HUVECs into intact tube-like structures and impaired the angiogenic capacity of HUVECs. Recent studies have found that FGF23 impairs endothelial function by activating the NF-κB signaling pathway, increasing oxidative stress to interfere with NO bioavailability, promoting HUVECs apoptosis and attenuating HUVECs migration^23^.

In addition, the results of this study showed that the expression levels of inflammatory factors in the serum of SONFH models were significantly higher than those in the normal group. Pyroptosis is a pro-inflammatory mode of programmed cell death, which is mediated by membrane porin (GSDM) and involves NOD-like receptor protein 3 (NLRP3) inflammasome and depends on cysteine aspartic acid (caspase)^24^. Also, NLRP3, GSDMD, and caspase-1 protein expression was found to be upregulated in our study, so we speculate that SONFH may activate the pyroptosis signaling pathway. The pyroptosis pathway in SONFH is a multifactorial and complex regulatory process. The current study found that NLRP3 inflammasomes mediated pyroptosis in BMSCs, thereby triggering a differentiation imbalance between osteoblasts and osteoclasts may play a key role in the development of SONFH^25,26^. NLRP3 inflammasomes activate capsase-1 to promote the maturation and secretion of IL-1β and IL-18, and Gasdermin D is cleaved to form peptides containing the N-terminal active domain of Gasdermin D, which enhances inflammation and mediates pyroptosis^27^. These results suggest inflammatory response and pyroptosis occur in SONFH. In addition, we also detected NLRP3, GSDMD, and caspase-1 activation in HUVECs, suggesting that inflammasomes are involved in the development of endothelial cell injury and exacerbate local ischemia and hypoxia in the necrotic femoral head to some extent, which is consistent with caspase-1-dependent inflammasomes activation in SONFH^28^. The results of this study also revealed that FGF23 silencing attenuated the inflammatory response of HUVECs and reduced the expression of NLRP3, GSDMD, and caspase-1. However, current studies on FGF23 in regulating pyroptosis signaling pathways are still limited. However, it has been suggested that inhibition of FGF23 improves inflammation in mice with chronic kidney disease in vivo and that blocking FGF23 activity could be a therapeutic target to reduce inflammation^29^.

Despite some limitations, this study elucidates the role and potential regulatory mechanisms of FGF23 in SONFH. Knockout mice were not used in this study. Compared to the study of Zhang et al.^30^, primary mouse BMECs were not isolated for investigating angiogenesis. In subsequent studies, it would be interesting to verify the effect of FGF23 on angiogenesis in primary mouse BMECs. In addition, bone remodeling is a balanced dynamic process that continuously carries out bone resorption and bone formation and requires the close cooperation of osteoblasts and osteoclasts. The role of osteoclasts in SONFH should be considered in the future studies.

This study demonstrates that FGF23 can regulate the pyroptosis signaling pathway, increase the release of inflammatory factors in SONFH, damage vascular endothelium, and inhibit osteogenic differentiation, thus affecting the development of SONFH, which provides a theoretical basis for FGF23 as a potential therapeutic target for SONFH.

### Supplementary Information

Supplementary Information.

## Data Availability

The datasets generated and analyzed during the present study are available from the corresponding author on reasonable request.
