# Supplementary material for: Fibroblast growth factor 23 inhibition attenuates steroid-induced osteonecrosis of the femoral head through pyroptosis
Source: Sci Rep. 2024 Jul 15;14:16270. doi: 10.1038/s41598-024-66799-z (PMC11251279; doi:10.1038/s41598-024-66799-z)

Figure 1A FGF23


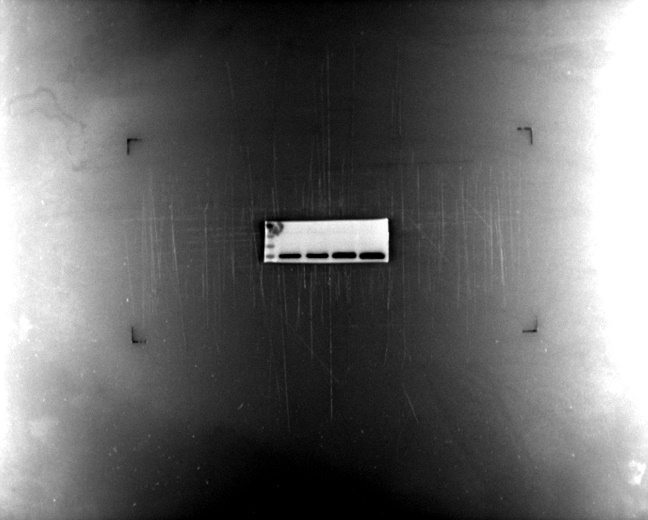


Figure 1A HIF-1α


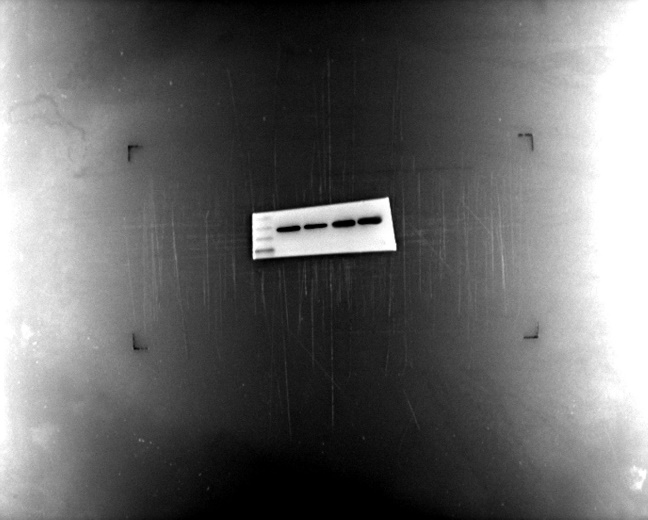


Figure 1A β-actin


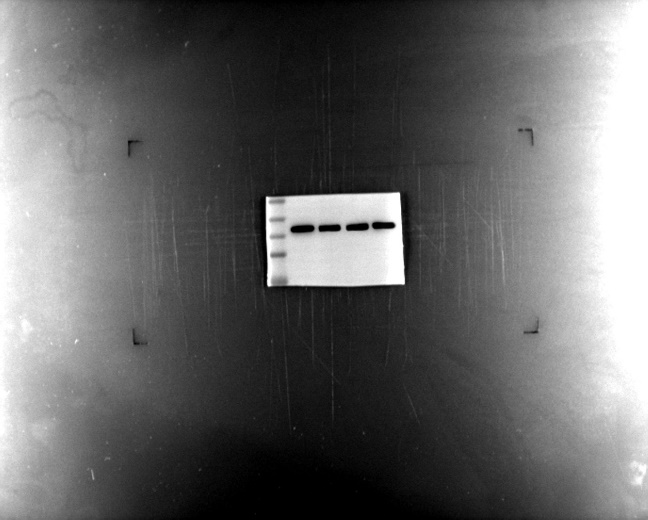


Figure 2C FGF23


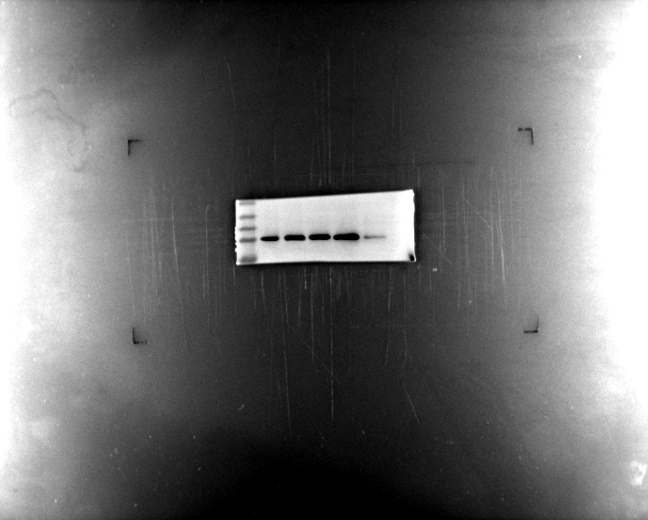


Figure 2C Runx2


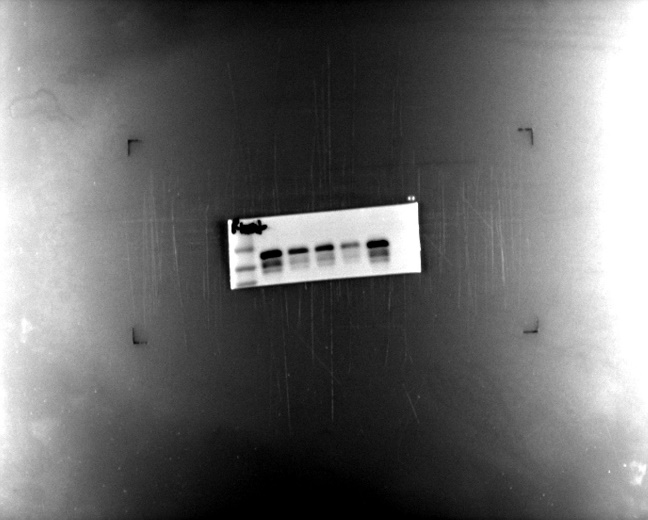


Figure 2C OCN


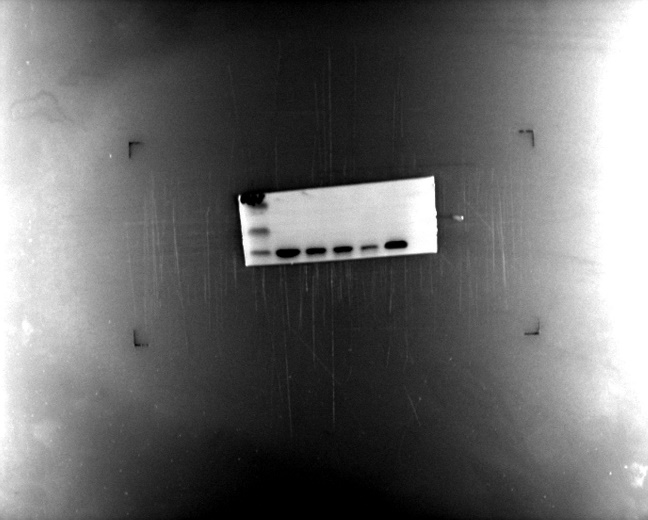


Figure 2C β-actin


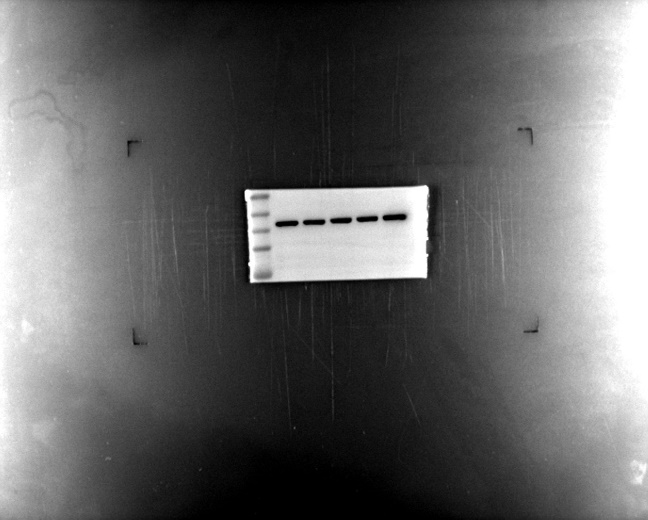


Figure 3H FGF23


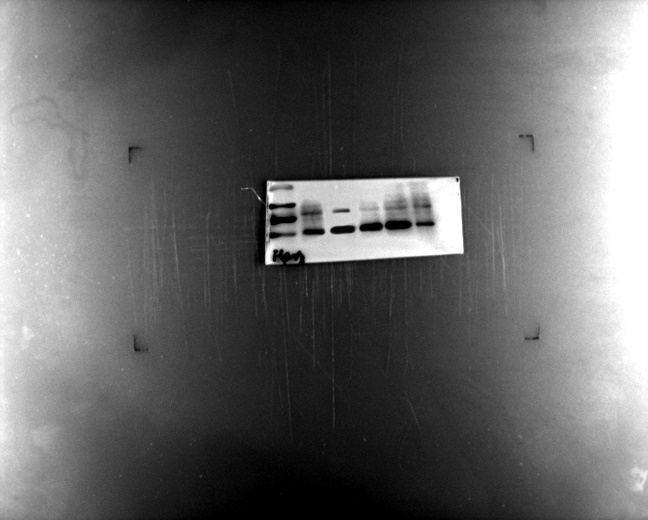


Figure 3H NLRP3


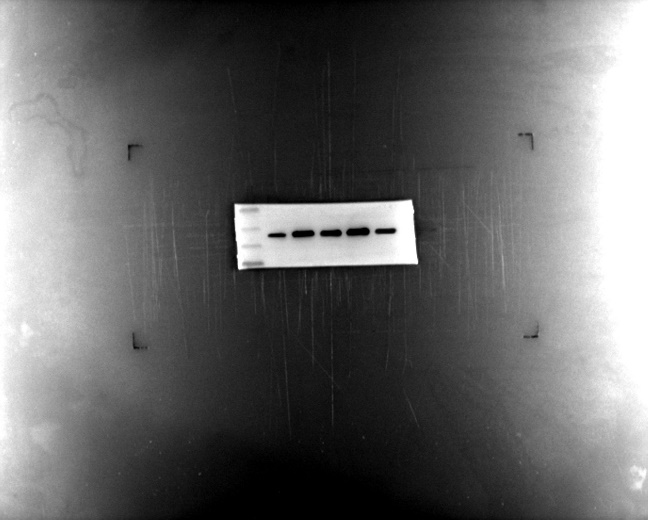


Figure 3H caspase-1


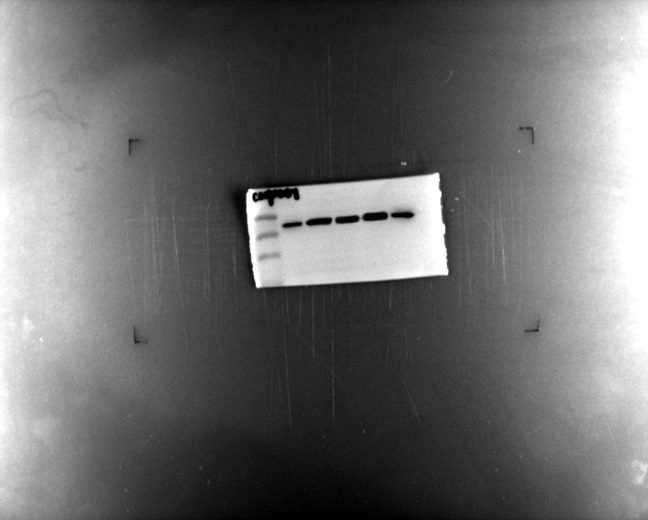


Figure 3H GSDMD


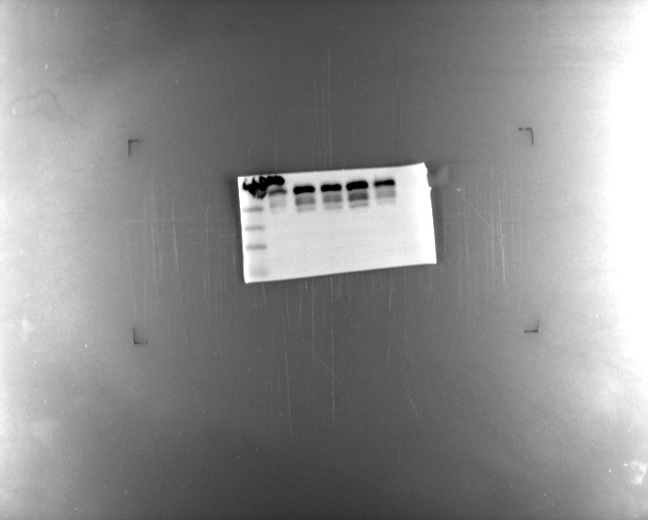


Figure 3H β-actin


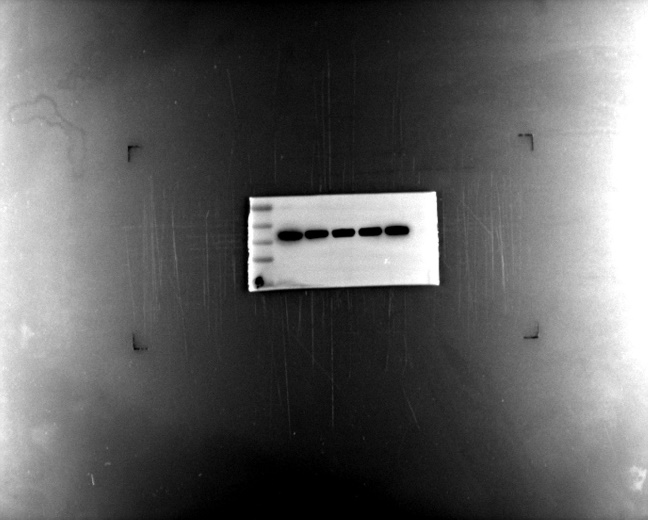


Figure 4C FGF23


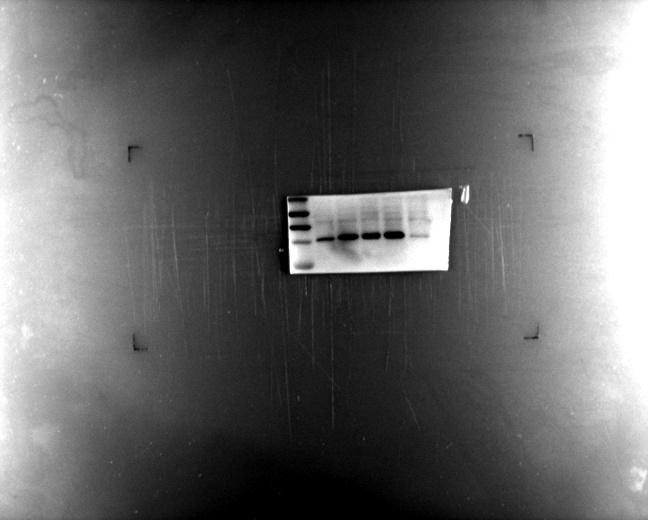


Figure 4C Runx2


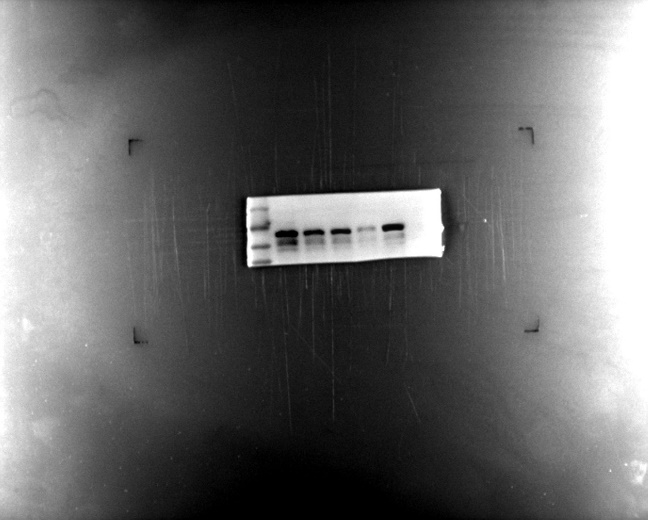


Figure 4C OCN


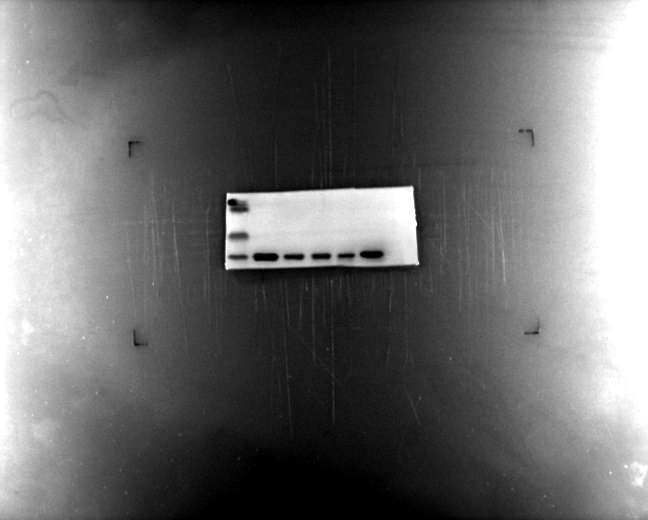


Figure 4C β-actin


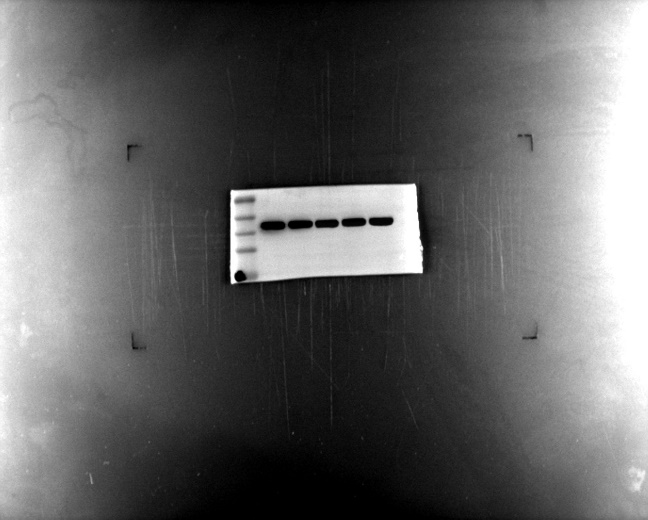


Figure 5A FGF23


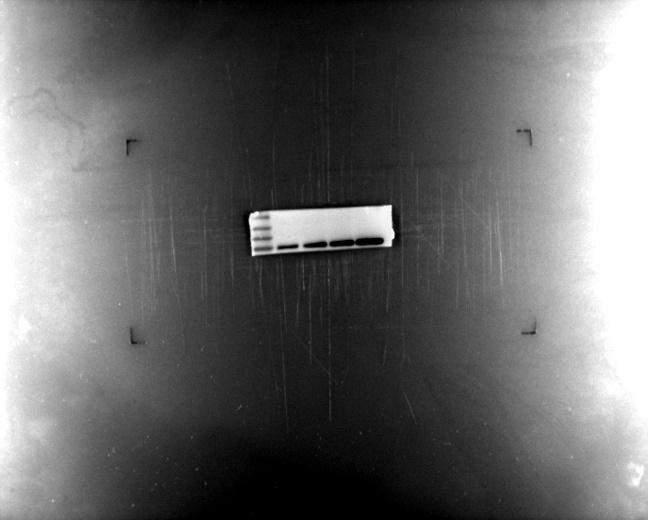


Figure 5A HIF-1α


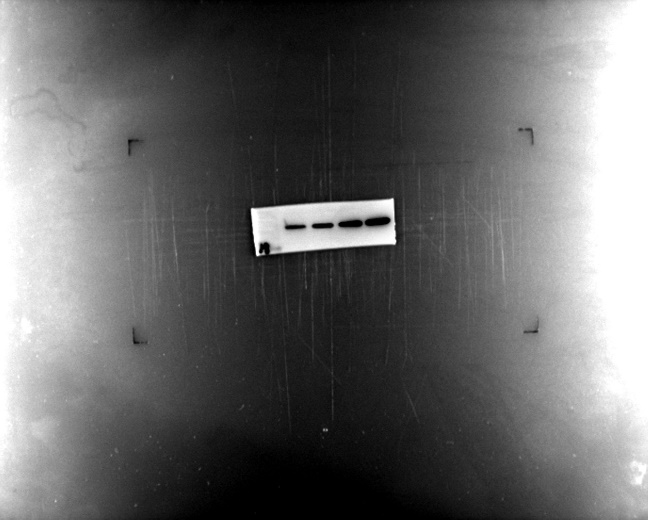


Figure 5A β-actin


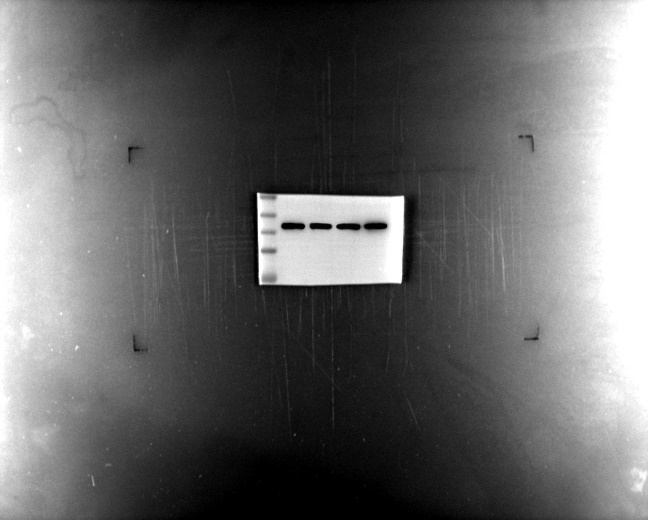


Figure 5C FGF23


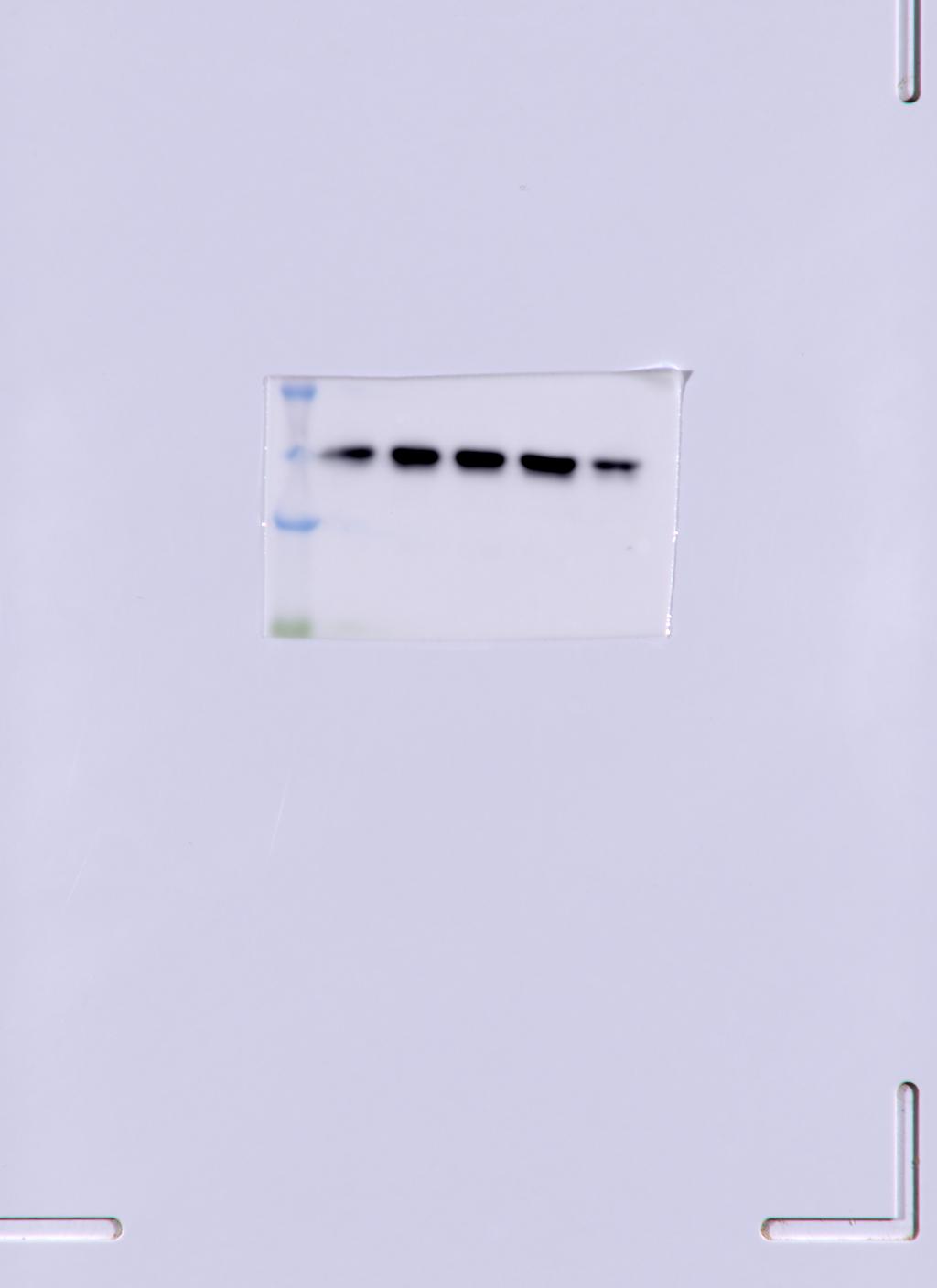


Figure 5C VEGF


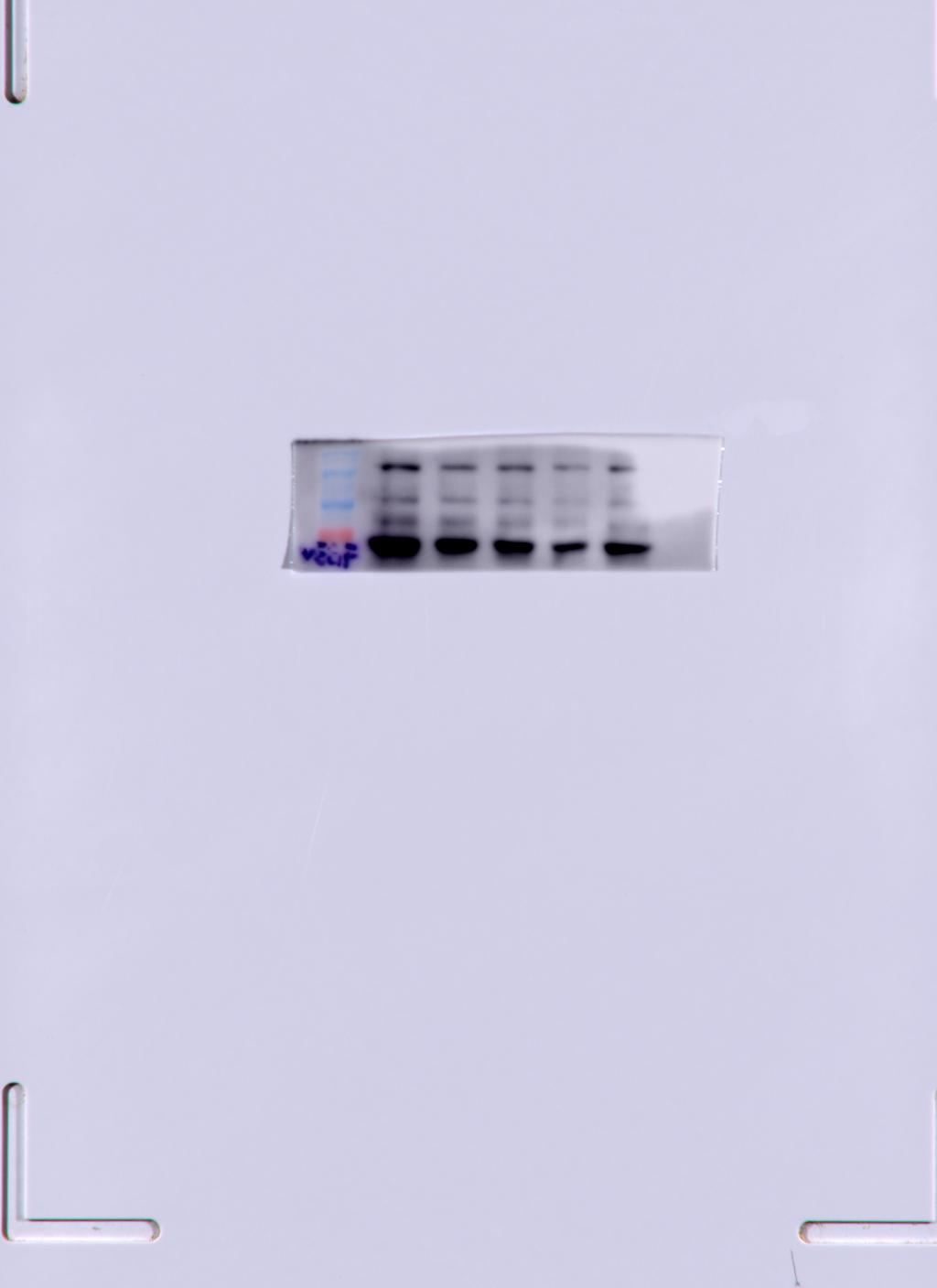


Figure 5C β-actin


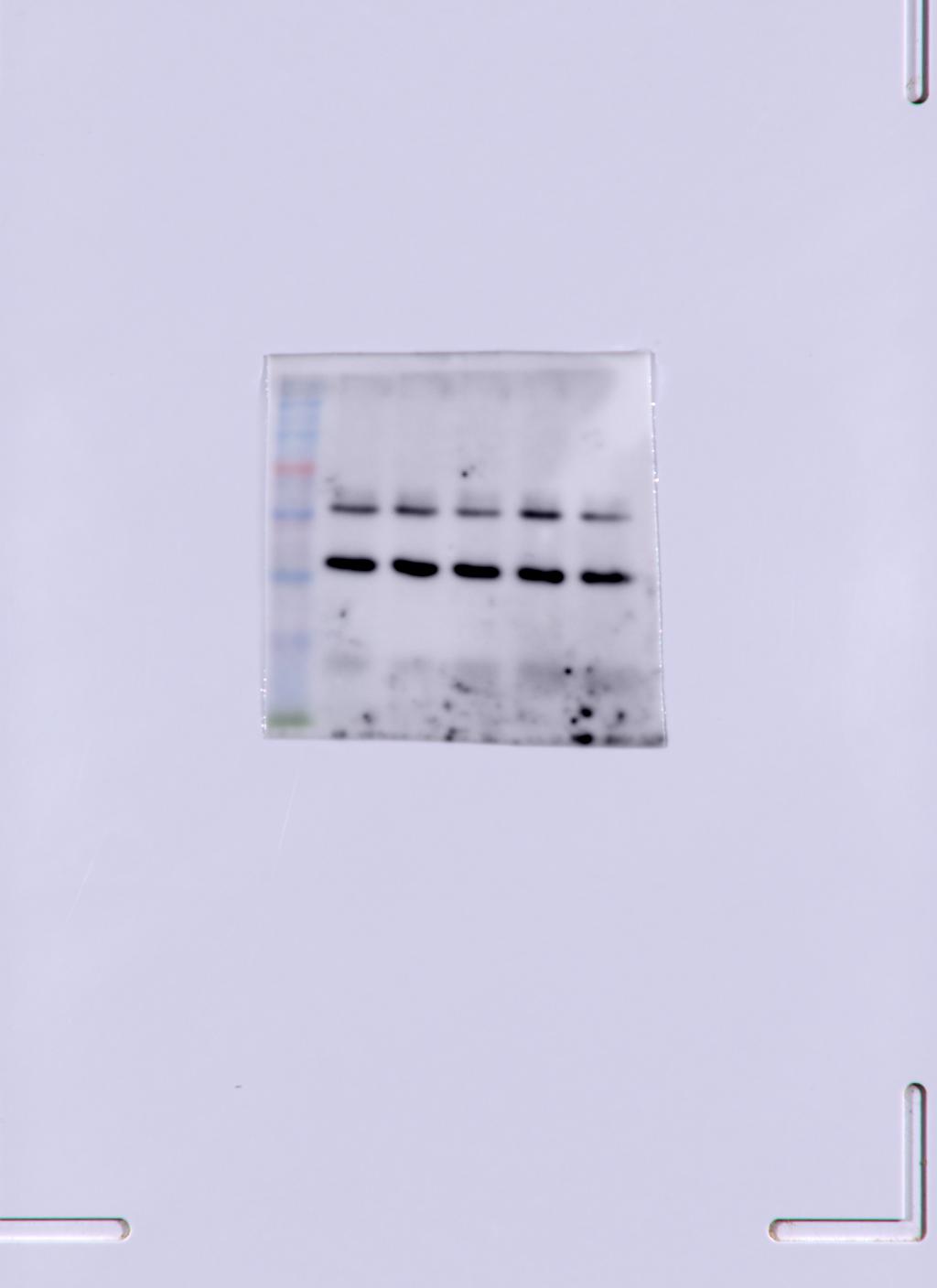


Figure 6F FGF23


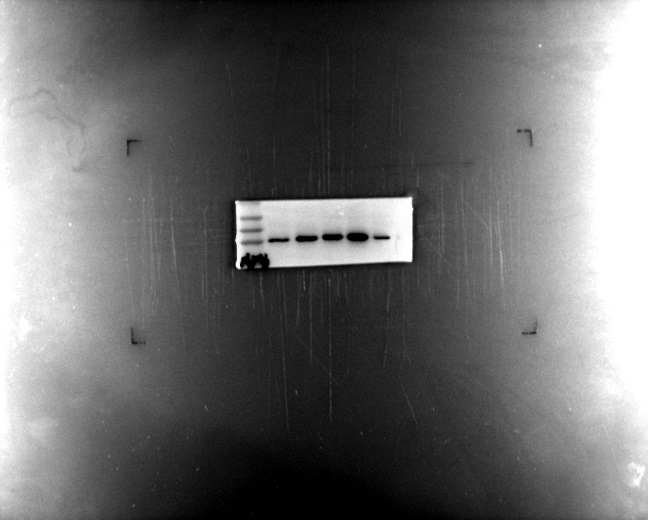


Figure 6F NLRP3


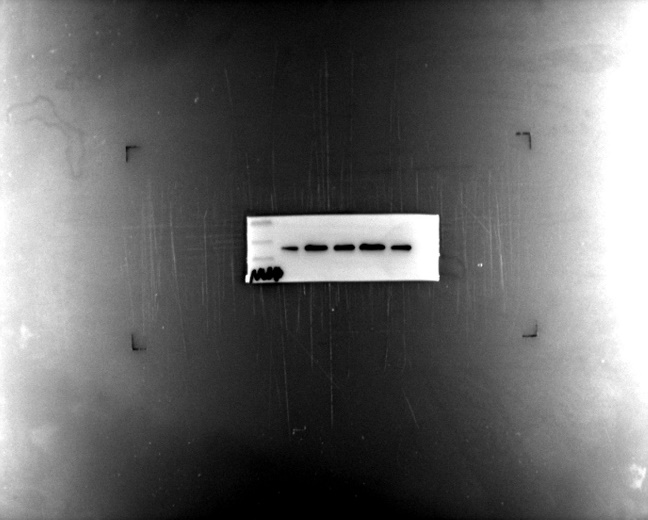


Figure 6F caspase-1


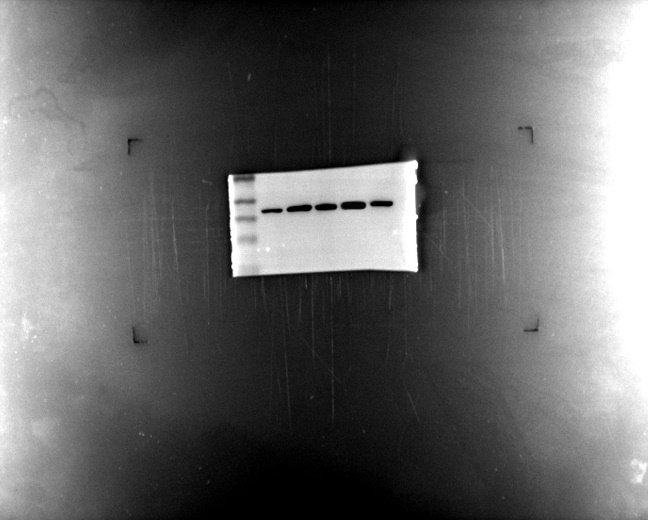


Figure 6F GSDMD


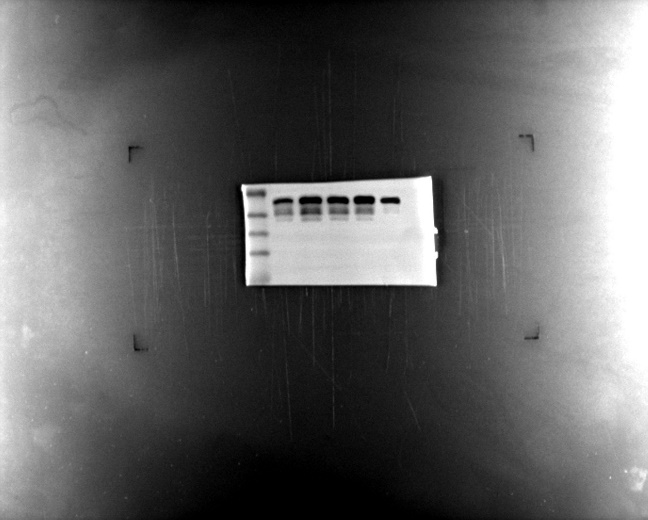


Figure 6F β-actin


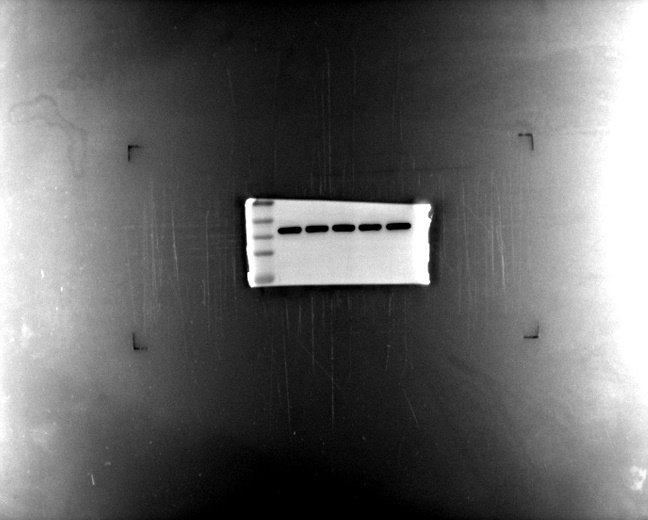

Supplement: Supplementary file 1 — Supplementary Information. [file 41598_2024_66799_MOESM1_ESM.docx]
